# Everything counts in sending the right message: science-based messaging implications from the 2020 WHO guidelines on physical activity and sedentary behaviour

**DOI:** 10.1186/s12966-020-01048-w

**Published:** 2020-11-04

**Authors:** Michelle L. Segar, Marta M. Marques, Antonio L. Palmeira, Anthony D. Okely

**Affiliations:** 1 The Sport, Health, and Activity Research and Policy Center, University of Michigan, 204. S. State St, Ann Arbor, MI 48109 USA; 2 ADAPT SFI Research Centre and Trinity Centre for Practice & Health Care Innovation, Trinity College Dublin, Dublin, Ireland; 3 CIDEFES, Universidade Lusófona, Campo Grande, 1740-024 Lisboa, Portugal; 4 Portugal & CIPER-FMH, Universidade Lisboa, Lisbon, Portugal; 5 Early Start, University of Wollongong, Wollongong, NSW 2522 Australia; 6 Illawarra Health & Medical Research Institute, Wollongong, NSW Australia

**Keywords:** Physical activity, Guidelines, Messaging, Framing, Communication, Self-determination theory

## Abstract

The World Health Organization (WHO) released the 2020 global guidelines on physical activity and sedentary behaviour. The new guidelines contain a significant change from the 2010 guidelines on physical activity for adults and older adults that has important implications for next-generation physical activity messaging: The removal of the need for aerobic activity to occur in bouts of at least 10 min duration. This change in the guidelines provides an opportunity to communicate in new ways that align with behavioural science, permitting physical activity communicators and promoters to better support people’s psychological needs, motivation, and ability to fit healthy levels of physical activity into their lives. The frames and messages we use to communicate about the guidelines matter because they influence whether activity is perceived as relevant, meaningful, and feasible – or not. When developing new physical activity communications there are some overarching principles, based on behavioural science, to keep in mind. Using established theory, this commentary aims to support the creation of more strategic frames and messages for increasing the value and integration of physical activity into daily living. Country-specific physical activity campaigns using these ideas will be discussed.

## Introduction

The World Health Organization (WHO) recently released the 2020 global guidelines on physical activity and sedentary behaviour [1, 2]. The primary goal of the guidelines is to summarize science showing the dose-response relationship between physical activity and health. As noted by Milton et al., [3] it is important to distinguish between the *actual* guidelines and *communicating about* the guidelines to the public. Thus, communication and messaging strategies are needed to disseminate the guidelines in ways that attract the public’s attention and also support the psychological needs associated with physical activity participation [4–6]. This commentary focuses on one core change in the new guidelines for adults and older adults that offers a valuable communication opportunity for next-generation physical activity messaging: The removal of the need for aerobic activity to occur in bouts of at least 10 min duration. This change opens the door to improve how we communicate about physical activity in a way that better aligns with motivation and behavioural science.

Despite decades of promoting the benefits of physical activity to the general population, activity levels are insufficient [7]. The significance of structural-level barriers that inhibit regular physical activity, such as unsafe neighbourhoods, cannot be overstated. Yet, as a field, we should acknowledge that traditional approaches to communicating about physical activity have not been effective. Communicating about physical activity has been based on the evidence about its relationship with health but it has often not been in accordance with the science about changing behaviour or communicating about health.

## Conventional threshold messages

To date, primary promotion and communication strategies for physical activity and its guidelines have been to communicate physical activity using *threshold messaging* – that is, the dose of physical activity needed to realize the desired physical and mental health benefits (e.g., amounts of activity in durations, intensities, etc.). There is an assumption underlying this use of threshold messaging that we now know is incorrect; namely, that increasing knowledge about the benefits of physical activity, and the participation requirements to achieve these benefits, does not sufficiently motivate people to participate. By their very nature, physical activity guidelines depend on people understanding and remembering them. While guidelines play an important role in our field, communications that emphasize the guidelines per se (e.g., recommended intensity level) are seemingly hard for people and even professionals to remember and understand.

For example, a large nationally representative survey investigated the proportion of US adults who were aware and knowledgeable of the 2008 Physical Activity Guidelines for Americans. Only 36% reported being aware of them, and less than 1% knew of the moderate-level physical activity recommendation emphasized in the guideline [8]. Research shows that even professionals who work in the fitness industry do not adequately grasp physical activity guidelines. Only 37% of fitness professionals at a national physical activity conference in Australia were able to recall the main message of the seminal US Surgeon General’s 1996 Report on Physical Activity and Health [9].

Threshold messages may also have unintended side effects. Consider that threshold messages are prescriptions of how long and the ways in which one should be active. In essence, they sanction only certain types and certain doses of physical activity. Repercussions from this type of messaging include people devaluing activities that do not achieve those criteria, feeling controlled and pressured by them, culminating in rigid, all-or-nothing thinking related to participation [6]; beliefs that inhibit the flexible thinking and response strategies that are associated with executive functioning [10] and sustainability of health-related behaviours [11, 12].

## Science-based message frames

The manner in which we communicate about a health behaviour *frames* it to the population and influences people’s perceptions about that behaviour and their decision-making related to performing that behaviour [13]. While there isn’t yet a consensus about the best way to frame physical activity [14], we do know that how we frame physical activity matters. The framing of physical activity influences whether people experience it as something that feels good (or bad) [15], is perceived as relevant to daily priorities [6], and is feasible to accommodate in one’s schedule [16].

The removal of the 10-min bout requirement for aerobic activity for adults and older adults in the 2020 WHO guidelines on physical activity and sedentary behaviour provides a strategic opportunity to reframe and communicate about physical activity in more adaptive and flexible ways. While there are many possible new messages that have the potential to communicate this significant change in the guidelines, this commentary primarily focuses on one: *Everything counts.*

It would be logical to assume that a message that democratizes all physical activity as valid would result in less physical activity than one that targets specific thresholds of duration and intensity. Yet research evaluating this communications question suggests otherwise. Mounting evidence shows that physical activity communications motivate *more* participation when we replace traditional threshold messages with ones that encourage more movement *of any kind* [17–19].

*Everything counts* as a message embodies this pivotal change in the WHO guideline; it gives people permission to be active with anyone, anywhere, for any amount of time, including walking or biking to work, gardening, cleaning, running after children, conducting walking meetings, dancing, and even just getting up from a desk to walk across the room. By legitimizing all physical activities and durations as valid and worth doing, such messaging engenders a new flexibility-boosting mindset and enables physical activity to become more feasible to fit into daily routines, which is especially important for caregivers and others who have many competing responsibilities and busy schedules [15]. Evidence from focus groups with individuals who participated in a physical activity intervention where *everything counts* was a key part of the curriculum highlights the adaptive nature of believing that *everything counts* when it comes to physical activity: “Before the class I wouldn’t go [to the gym] unless I had a good hour... after [the class] I would go, even if I could only take a 30- or 15-minute walk around the track” [20].

The pragmatic benefits from believing that *everything counts* when it comes to being physically active are clear. Yet established behavioural theory can help us better understand the potential of an all-inclusive physical activity message to transform people’s physical activity mindset and behaviour.

## Everything counts: theoretical rationale

A message such as *everything counts* aligns with self-determination theory (SDT), a prominent macro-theory of human motivation. SDT emphasizes the role of social environments in supporting what are considered to be key psychological needs. Ryan and Deci have identified these three needs as 1. Autonomy – having a sense of choice and self-direction about one’s behaviour, 2. Competence – feeling effective in one’s interactions with the environment and in performing a behaviour, and 3. Relatedness – feeling accepted and respected by and connected to other people [21]. Research suggests that SDT-based interventions support psychological needs and help facilitate the internalization of motivation, eliciting short- and long-term behaviour change across many different behaviours, including dietary change, physical activity, weight management, and medication adherence [22, 23].

Adopting an all-inclusive message such as *everything counts* to promote physical activity supports all three psychological needs within SDT. It fosters *competence* because it transcends real or perceived barriers to being physically active related to time, energy, or skills. If *everything counts*, anyone can do it.

*Everything counts* cultivates *relatedness*. No longer is physical activity relegated to the solitary domain of individual fitness; it becomes a way that people can connect with important others *while being* active. Caregivers are often not active because they view physical activity as competing with their family’s needs [16, 24]. When *everything counts*, however, they have permission to see physical activity as supporting their family because it becomes a valid way to spend time *with* their families through activities of daily living, play, and transport.

*Everything counts* is also inherently *autonomous*. It removes the need to meet specific criteria that might feel controlling. With an *everything counts* mindset, people can choose activities that they desire or feel valuable to do. These benefits synergistically combine to result in physical activity becoming more intrinsically motivating, something that is considered important for creating sustainable changes in behaviour.

*Everything counts* is inclusive of everyone with any body type and physical capabilities. If being active outside is not safe, it legitimizes choosing to move inside. Ultimately, *everything counts* takes the pressure off by giving people permission to be active anywhere, anytime, and in accordance with their own wants, needs, and circumstances [25].

While any message or campaign needs a formal evaluation [3], we propose that physical activity communications and messages will have the greatest potential to motivate sustained physical activity participation if they support people’s autonomy, competence, and relatedness toward being physically active [22]. By using the message *everything counts*, we can help the population internalize this new belief and flexible mindset. But *everything counts* is just one of myriad potential messages that can embody this change and support autonomy, relatedness, and competence.

## Many possible messages

Several countries have created population-based physical activity campaigns using all-inclusive physical activity messages that support people’s psychological needs. For example, in the United States, the *Move Your Way* campaign targets individuals across the lifespan [26]. Similar to *everything counts*, *Move Your Way* cultivates competence by explicitly giving people license to move in ways that they feel comfortable and capable of doing. It is inherently autonomous because it explicitly advocates that people be active in the ways that they desire and choose. Relatedness and autonomy are jointly supported through images that accompany the campaign of people enjoying being active together.

Another needs-supporting campaign was designed to promote physical activity to adult women in England [27]. Sport England’s *This Girl Can* campaign proclaims “there’s no right way to get active.” This specific message endorses an all-inclusive definition of valid physical activity. It is accompanied by images that feature women of different ages participating in a wide range of activities, emphasizing that when it comes to being physically active, everything counts.

Portugal’s physical activity campaign “physical activity is calling you” (i.e., *A atividade física chama por si*) offers another example of how communications can be designed to support people’s needs in relation to being physically active [27]. This campaign’s featured video shows a large variety of physical activities in addition to explicitly asking the viewer “What is *your* activity?” Similar to the *Move Your Way* campaign, Portugal’s campaign invites each individual to autonomously choose their own activity based on their preferences.

To effectively communicate physical activity guidelines it is important to also communicate to the professional stakeholders and advocates who will disseminate the guidelines to the public [3, 28]. Yet, even professionals who work in the field do not know or cannot recall the specific details within physical activity guidelines [9]. Thus, even messages to professional stakeholders about how to communicate physical activity guidelines also need to be easy to remember and internalize. Messages like *everything counts* meet that need.

## Conclusion

By removing the requirement for aerobic activity to occur in bouts of at least 10 min duration, the 2020 WHO guidelines on physical activity and sedentary behaviour permit our field to communicate about physical activity with next-generation messages that offer a menu of possibilities from which people can autonomously choose and feel competent doing, alone or with others. Fundamentally, the 2020 guidelines offer our global community a perfect opportunity to communicate new, simple, scientifically supported concepts and messages that are easy understand, easy to remember, and able to be put into practice in different ways across populations and contexts. Everything counts in sending the right message.

## Data Availability

Not applicable.
